# Road Trauma in Teenage Male Youth with Childhood Disruptive Behavior Disorders: A Population Based Analysis

**DOI:** 10.1371/journal.pmed.1000369

**Published:** 2010-11-16

**Authors:** Donald A. Redelmeier, William K. Chan, Hong Lu

**Affiliations:** 1 Department of Medicine, University of Toronto, Toronto, Canada; 2 Clinical Epidemiology Program, Sunnybrook Health Sciences Centre, Toronto, Canada; 3 Institute for Clinical Evaluative Sciences in Ontario, Ontario, Canada; 4 Patient Safety Service, Sunnybrook Research Institute, Toronto, Canada; Research Center INSERM U897, France

## Abstract

**Background:**

Teenage male drivers contribute to a large number of serious road crashes despite low rates of driving and excellent physical health. We examined the amount of road trauma involving teenage male youth that might be explained by prior disruptive behavior disorders (attention deficit hyperactivity disorder, conduct disorder, oppositional defiant disorder).

**Methods and Findings:**

We conducted a population-based case-control study of consecutive male youth between age 16 and 19 years hospitalized for road trauma (cases) or appendicitis (controls) in Ontario, Canada over 7 years (April 1, 2002 through March 31, 2009). Using universal health care databases, we identified prior psychiatric diagnoses for each individual during the decade before admission. Overall, a total of 3,421 patients were admitted for road trauma (cases) and 3,812 for appendicitis (controls). A history of disruptive behavior disorders was significantly more frequent among trauma patients than controls (767 of 3,421 versus 664 of 3,812), equal to a one-third increase in the relative risk of road trauma (odds ratio  =  1.37, 95% confidence interval 1.22–1.54, *p*<0.001). The risk was evident over a range of settings and after adjustment for measured confounders (odds ratio 1.38, 95% confidence interval 1.21–1.56, *p*<0.001). The risk explained about one-in-20 crashes, was apparent years before the event, extended to those who died, and persisted among those involved as pedestrians.

**Conclusions:**

Disruptive behavior disorders explain a significant amount of road trauma in teenage male youth. Programs addressing such disorders should be considered to prevent injuries.

*Please see later in the article for the Editors' Summary*

## Abstract 2

Donald Redelmeier and colleagues conducted a population-based case-control study of 16-19-year-old males hospitalized for road trauma or appendicitis and showed that disruptive behavior disorders explained a significant amount of road trauma in this group.

## Editors' Summary

**Background:**

In the latest World Health Organization (WHO) global burden of disease list, road traffic crashes are currently ranked eighth but are predicted to take fourth place by 2030 (by which time, road traffic deaths are likely to increase by more than 80% in developing countries and to decrease by nearly 30% in industrialized countries.) Every year, road traffic crashes kill an estimated 1.2 million people world-wide and injure or disable a further 20–60 million. Furthermore, the economic consequences of road traffic crashes account for about 2% of the gross national product of the entire global economy.

90% of road traffic deaths occur in developing countries where pedestrians, cyclists, and users of two-wheel vehicles (scooters, motorbikes) are the most vulnerable. In industrialized countries, teenage male drivers are the single most risky demographic group, with an incidence of road traffic crashes of twice that of the population average. Also, male teenagers are sometimes a hazard to other road users and contribute to more fatalities in older pedestrians than older drivers. Furthermore, teenage male drivers involved in serious crashes can have ongoing health care needs but are often resistant to standard road safety advice.

**Why Was This Study Done?:**

Previous studies have suggested that disruptive behavior disorders might contribute to the risk of road traffic crashes in male teenagers but methodological problems with these studies make these results unclear. Given the importance of this topic, authorities have called for more research into the full range of behavioral disorders and relevant populations. This study attempted to avoid the methodological problems of previous studies and to rigorously assess whether disruptive behavior disorders predispose male teenagers to road traffic crashes.

**What Did the Researchers Do and Find?:**

The researchers conducted a 7-year population-based case-control study in Ontario, Canada of consecutive male teenagers aged between 16 and 19 years who were admitted to a hospital due to a road traffic crash, including those who were pedestrians. For the controls, the researchers used consecutive males in the same age range who were admitted to the same hospitals during the same time interval for acute appendicitis (which is common and generally unrelated to traumatic injury). For each participant in the study, the authors used universal health care databases in Canada's single-payer health care system to identify relevant psychiatric diagnoses (attention deficit hyperactivity disorder, conduct disorder, and oppositional defiant disorder) during the decade before admission.

During the study period, 3,421 male teenagers were admitted to hospital as the result of a road traffic crash and 3,812 male teenagers were admitted to hospital for appendicitis. A history of disruptive behavior disorders was significantly more frequent among male teenagers admitted for road traffic crashes than controls (767 of 3,421 versus 664 of 3,812) giving an odds ratio 1.37. This higher risk was still present after the researchers adjusted for possible confounding factors (such as age, social status, and home location) and accounted for about one-in-20 road traffic crashes, including male teenagers who had died and those involved as pedestrians.

**What Do These Findings Mean?:**

The results of this study suggest that disruptive behavior disorders explain a significant amount of road traffic crashes experienced in male teenagers. Overall, attention deficit hyperactivity disorder, conduct disorder, and oppositional defiant disorder are associated with about a one-third increase in the risk of a road traffic crash (which is similar to the relative risk among individuals treated for epilepsy.) As in previous studies in this area, some methodological problems may affect the interpretation of these findings. As this study did not document who was “at fault,” an alternative interpretation might be that behavioral disorders impair a teenager's ability to avoid a mishap initiated by someone else. Most importantly, the observed increase in risk as pedestrians indicates that male teenagers who abstain from driving do not escape the danger of road traffic crashes.

The researchers stress that any increased risk of road traffic crashes associated with disruptive behavior disorders in male teenagers does not justify withholding a driver's license, especially as many such disorders can be effectively treated or, indeed, because it does not address the issue of the increased risk for those teenagers who were pedestrians. Instead, they suggest that disruptive behavior disorders could be considered as contributors to road traffic crashes—analogous to seizure disorders and some other medical diseases. Therefore, greater attention by primary care physicians, psychiatrists, and community health workers might be helpful since interventions can perhaps reduce the risk including medical treatments and avoidance of distractions.

**Additional Information:**

Please access these Web sites via the online version of this summary at http://dx.doi.org/10.1371/journal.pmed.1000369.

- The World Health Organization has information on road traffic crashes
- The US National Institutes of Health has information about behavior disorders in children as well as UK-based Kids Development
- The Ontarion Ministry of Transportation has information on annual roadway collisions in Ontario

## Introduction

Road crashes are a common cause of death, disability, and property loss throughout the world equating to around 2% of the gross national product of the entire global economy [1]. Teenage male drivers are the single most risky demographic group, with an incidence twice the population average [2]–[4]. Teenage male drivers involved in serious crashes can also have especially devastating outcomes related to ongoing needs for health care as well as foregone future productivity [5]. In addition, young drivers are sometimes a hazard to other road users and contribute to more fatalities in older pedestrians than older drivers themselves [6]–[8]. Unfortunately, teenage male drivers are often remarkable in risk attitudes and resistant to standard safety advice [9],[10].

Safety regulation is a countermeasure for preventing road trauma in drivers. For example, most countries prohibit driving before age 16 y [11]–[13]. Some regions have further restrictions using graduated licensing programs that disallow young drivers from night driving, high-speed roadways, and additional hazardous settings [14]–[16]. Regulations based on age are often supplemented by restrictions related to diabetes mellitus, seizure disorders, or other medical illnesses [17]. Regulations have generally not been feasible, however, for curbing many forms of driver inattention contributing to crashes [18]. As a consequence, road trauma is a common cause of death and disability until about age 40 y and indicates that prevailing regulations (and self-restrictions) are insufficient [19],[20].

Past research has suggested that disruptive behavior disorders might contribute to the risk of serious road trauma in teenage males [21]–[24]. The evidence suggests that these disorders are frequent during childhood and adolescence, characterized by impulsivity with rule infringement, and identified in some cases of trauma [25]–[29]. Past studies, however, raise uncertainties because of small sample size, referral bias, surrogate outcomes, inadequate controls, and self-report bias [30],. Authorities, therefore, have called for more research stressing that the full range of disorders is understudied and misunderstood [32]–[34]. The purpose of this study was to avoid such biases and assess how much disruptive behavior disorders predispose teenage males to serious road trauma.

## Methods

### Setting

Ontario was one of the largest Canadian provinces in 2005 (study midpoint) and had 16,599.8 km of roadway, 246 acute care hospitals, 766 roadway fatalities, and a total population of 12,160,280 individuals (of whom 664,865 were between age 16 and 19 y) [35]–[40]. In this study we used universal health care databases in Canada's single-payer health care system to conduct a population-based retrospective case-control analysis of teenagers involved in serious road trauma between April 1, 2002 and March 31, 2009, representing all years available for analysis. These databases have been validated in past medical research and prior analyses have used these databases to provide measures of relative risks, absolute risk, and attributable risk [41]–[43]. The study was approved by the Research Ethics Board of Sunnybrook Health Sciences Center.

### Patients

Cases were identified as consecutive males between age 16 and 19 y admitted to an acute care hospital for motor vehicle related trauma (codes V01 to V99). Controls were identified as consecutive males in the same age range admitted to the same hospitals during the same time interval for acute appendicitis (codes K35 to K38). We chose this control condition because it was frequent, clearly coded in hospital records, generally unrelated to traumatic injury, not known to protect against other childhood disorders, and has served as a standard for other research [44]–[49]. We excluded teenage girls from both groups to avoid Simpson's paradox (a spurious association created by loading on a null-null position) since this group has much lower rates of crash involvement [50],[51].

### Driving

We directed special attention to distinguish different patterns of road trauma. In accord with prior research [52], we characterized each case using four categories: driver, passenger, pedestrian, and miscellaneous. The pedestrian category also included other vulnerable road users (e.g., bicyclists) and the miscellaneous category included unusual events (e.g., skateboards and snowmobiles). We also stratified trauma severity according to medical management by following each patient during hospitalization for surgery, critical care treatment, or death. The available databases contained no data on crash hour, other people in the same collision, or at-fault determinations by police.

### Disorders

We focused on selected disorders relevant to childhood, defined by the Diagnostic and Statistical Manual of Mental Disorders, and associated with inattention or distraction. The specific disorders were attention deficit hyperactivity disorder, conduct disorder, and oppositional defiant disorder (codes 312 to 314) [53]–[56]. Others use the term “attention deficit related disorders,” “childhood behavioral disorder,” or “externalizing disorder” to denote these conditions since combinations are frequent, exact diagnoses are not always possible, and diagnostic criteria change over time [57]–[60]. We did not examine internalizing disorders characterized by anxiety or excess deliberation such as social phobia, obsessive compulsive disorder, or anorexia nervosa.

### Ascertainment

For both cases and controls, we searched outpatient database records for a decade prior to admission to identify any disruptive behavior disorder diagnosed earlier (after age 5 y). This strategy assured that ascertainment was blind to outcome status, free of reporting bias, and avoided reverse-causality artifacts [61],[62]. This strategy also allowed us to examine complex combinations occurring together, such as attention deficit hyperactivity disorder combined with substance abuse or another neuropsychiatric condition. These methods have been validated extensively in past research in Canada's single-payer universal health insurance system and were conducted using privacy safeguards of the Institute for Clinical Evaluative Sciences in Ontario [63].

### Severity

We used multiple measures to gauge the severity of each disorder because the available records did not contain results from psychological testing. Age at onset of the disorder was defined as the date first diagnosed by a physician. Intensity of care was defined as the mean number of physician visits per year as well as the total years of treatment for the disorder. Case complexity was also characterized by the total number of visits to a board-certified psychiatrist as well as any mention of substance abuse, learning disorders, depression, personality disorders, epilepsy, movement disorders, or mental developmental delay. The available databases did not contain information on drug therapy, patient adherence, social services, school performance, or special resources.

### Validation

We conducted secondary analyses to examine the robustness of our findings. We used three separate tracer conditions to explore whether the risk associated with psychiatric disorders was distinct and not shared by other childhood illnesses; namely, asthma (code 493), contact dermatitis (code 692), and otitis media (codes 381 to 382). The purpose of these analyses was to check for the absence of an association where no association would be expected [64]. In addition, we stratified patients according to their short-term medical outcomes; namely, those patients who had a prolonged length of stay (>7 d), critical care unit admission, surgical operation, or death. The purpose of these analyses was to check how findings extended across a spectrum of increasing trauma severity.

### Statistics

The primary analysis examined the prevalence of prior disruptive behavior disorders among cases involved in a crash compared to controls not involved in a crash using an unpaired chi-square test congruent with the case-control design [65]. Logistic regression was used to further quantify associations using odds ratios to adjust for imbalances in demographic characteristics (age, social status, home location) and prior neuropsychiatric diagnoses (each coded separately). Logistic regression was also used to explore additional risk factors among patients positive for a prior disorder. Calculations of attributable risk and attributable fraction were conducted using population-based methods [66].

## Results

During the 7-y interval a total of 3,421 emergency admissions occurred for 3,421 teenage male patients involved in road trauma over 146 hospitals and 1,445 attending physicians. We observed no major trends over the years. The typical patient had a mean age of 17.6 y, was a driver (71%, *n = *2,443), and had been traveling on a public roadway (61%, *n = *2,070). Almost all had visited a physician during the decade before hospital admission (98%, *n = *3,356). A large number lived in rural areas (29%, *n = *978), crashed on a weekend (39%, *n = *1,334), involved another vehicle (40%, *n = *1,368), and presented in the summer (37%, *n = *1,277). In comparison, 3,812 control patients were admitted for acute appendicitis over the same interval and same hospitals (Table 1).

**Table 1:** Patient characteristics.

Patient Characteristics	Subcategory	Trauma(*n = *3,421)	Control(*n = *3,812)
Age (y)	16–17	1,667 (49)	1,996 (52)
	18–19	1,754 (51)	1,816 (48)
Socioeconomic status^a^	Lowest	595 (17)	676 (18)
	Next lower	974 (20)	719 (19)
	Middle	698 (20)	744 (19)
	Next higher	721 (21)	770 (20)
	Highest	702 (20)	891 (23)
Home location^a^	Urban	2,438 (71)	3,207 (84)
	Rural	978 (29)	600 (16)
Prior admissions	Any in past 3 y	275 (8)	212 (6)
Prior emergency visits	Number in past 3 y	2.3±2.8	1.8±2.4
Prior clinic visits	Number in past 3 y	7.1±7.6	7.8±7.5
Time since last clinic visit (d)		237±254	201±238
Season of year	Spring	720 (21)	942 (25)
	Summer	1,277 (37)	1,027 (27)
	Autumn	883 (26)	970 (25)
	Winter	541 (16)	873 (23)
Day of admission^b^	Weekday	2,087 (61)	2,833 (74)
	Weekend	1,334 (39)	979 (26)

Data are count (percentage) except where noted as mean±standard deviation.

a May not sum to 100% owing to rounding and missing values.

b Saturday and Sunday denote weekend.

A history of a prior disruptive behavior disorder was significantly more common among cases than controls (Table 2). Based on the case-control design, this association was equal to a one-third increase in the risk of road trauma (odds ratio 1.37, 95% confidence interval 1.22–1.54, chi-square  =  28, *p*<0.001). The increased trauma risk was evident for those with a history of attention deficit hyperactivity disorder, a history of other disruptive behavior disorder, or both types of histories clustered together (Table 3). In contrast, no adverse association was observed with a history of asthma (odds ratio 0.97, 95% confidence interval 0.87–1.07). Similarly, no major association was observed with a history of contact dermatitis (odds ratio 1.06, 95% confidence interval 0.93–1.20) or otitis media (odds ratio 1.10, 95% confidence interval 1.01–1.22).

**Table 2:** Prior diagnoses.

Prior Diagnoses	Trauma	Control
Any disruptive behavior disorder^a^	767 (22)	664 (17)
Attention deficit hyperactivity disorder (code 314)	402 (12)	344 (9)
Other disruptive behavior disorder (code 312, 313)	625 (18)	531 (14)
Both disorders	260 (8)	211 (5)
Among those with a disorder		
Age at first psychiatric visit	10±3	10±3
Total days from first to last psychiatric visit	1,013±1105	903±1,097
Age at latest psychiatric visit (y)	13±3	13±3
Total *n* psychiatric visits	7.5±13.3	6.9±10.7
Total *n* specialist psychiatrist visits^b^	3.8±14.6	4.3±13.5
Days since last psychiatric visit to admission	1,608±1037	1,647±1,068
Other neuropsychiatric comorbidity	766 (22)	746 (19)
Substance abuse (code 303, 304, 305)	169 (5)	112 (3)
Learning disorder (code 315)	139 (4)	122 (3)
Depression (code 296, 311)	172 (5)	150 (4)
Personality disorder (code 301)	62 (2)	68 (2)
Epilepsy (code 345)	67 (2)	62 (2)
Movement disorder (code 307)	340 (10)	380 (10)
Mental developmental delay (code 317, 318, 319)	25 (1)	21 (1)
Unrelated medical illnesses		
Asthma (code 493)	939 (27)	1070 (28)
Contact dermatitis (code 692)	504 (15)	536 (14)
Otitis media (code 381, 382)	1,445 (42)	1,517 (40)

Data are count (percentage) except where noted as mean ± standard deviation.

a Codes are ICD9 codes extracted from outpatient records in decade prior to admission.

b Denotes outpatient visit to board-certified psychiatrist.

**Table 3:** Crash risk according to cluster of disruptive behavior disorders.

Disruptive Behavior Disorder	Odds Ratio a	Lower 95%ConfidenceInterval	Upper 95%ConfidenceInterval
ADHD or Other or both	1.37	1.22	1.54
Any mention of ADHD	1.34	1.15	1.56
Any mention of Other	1.38	1.21	1.57
Sole mention of ADHD	1.20	0.94	1.52
Sole mention of Other	1.30	1.11	1.53
Both ADHD and Other	1.40	1.16	1.69

ADHD, attention deficit hyperactivity disorder (code 314); Other, other disruptive behavior disorders (code 312, 313).

a From univariate analysis correlating cluster with increased crash risk.

**Figure 1:**
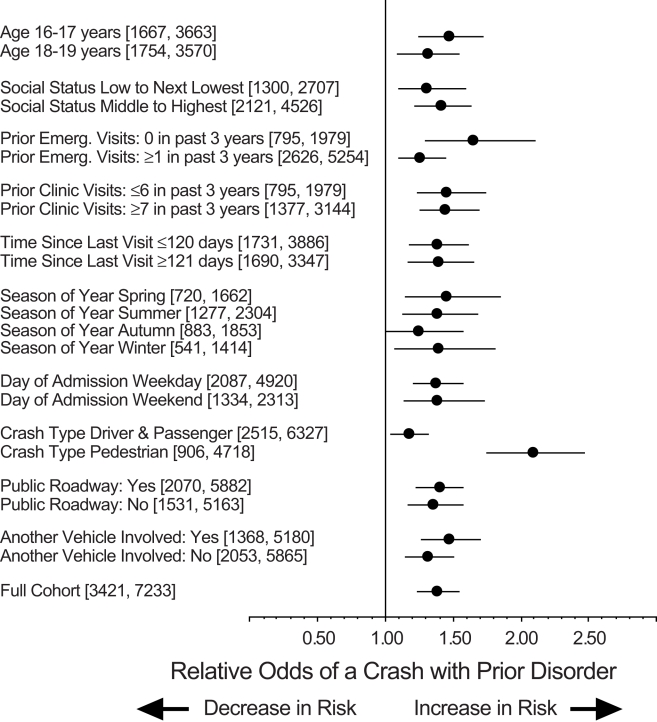
Crash risk in different subgroups. Each analysis examines correlation of a history of a disruptive behavior disorder with higher relative risk of a crash. Event counts and sample size for each subgroup appear in square brackets. Results expressed as odds ratio (solid circle) and 95% confidence interval (horizontal line). Analyses of crash type, public roadway, and other vehicle involvement based on all controls. Results for full cohort appear at bottom and show odds ratio of 1.37 with 95% confidence interval 1.22–1.54.

The association of disruptive behavior disorders and increased risk of trauma was consistent for patients with different characteristics (Figure 1). Most subgroups overlapped the primary analysis and no subgroup showed a contrary pattern. The increased risk was apparent in crashes that did or did not involve another vehicle and was not accentuated for crashes during weekends or summer months (unlike crashes due to alcohol). The largest increase was observed in the subgroup analysis of pedestrians that showed a doubling of risk. Multivariable analysis adjusting for demographic characteristics (age, social status, home location) and neuropsychiatric comorbidities showed a somewhat larger increase in the risk of road trauma associated with prior disorders (odds ratio  =  1.38, 95% confidence interval 1.21–1.56, *p*<0.001).

**Figure 2:**
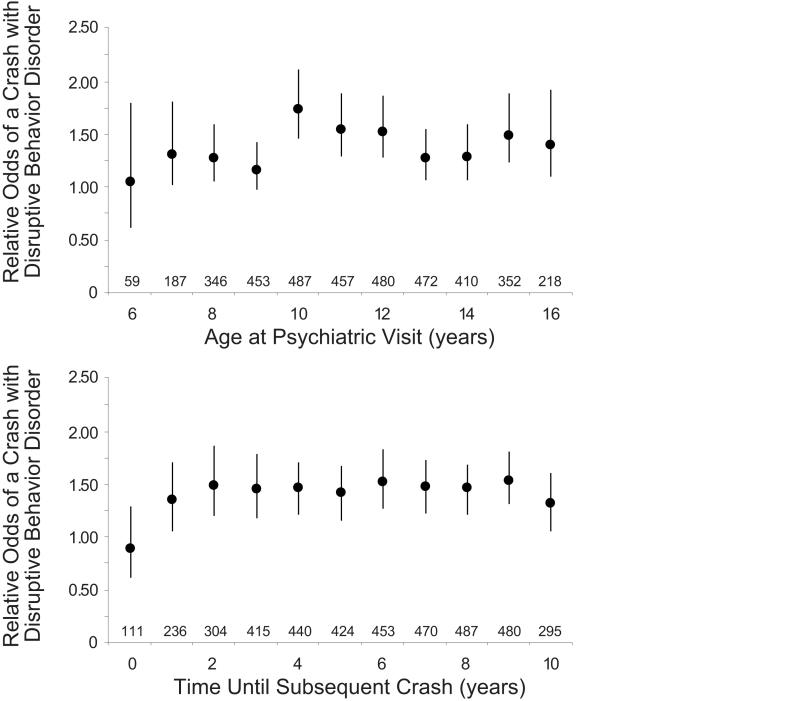
Timing of prior psychiatric visit and crash risk. Each analysis examines correlation of psychiatric visit for a disruptive behavior disorder with higher relative risk of a crash. Estimates calculated in 1-y intervals based on whether patient had any psychiatric visit during corresponding year. Upper panel for patient age at visit and lower panel for time from visit to subsequent crash. Numbers above horizontal axis denote count of patients with a visit during interval. Findings expressed as odds ratio (solid circle) and 95% confidence interval (vertical line). Results show increases years before the crash and potential maximum at age 10 y.

Two aspects of the patient history accentuated the observed association as independent risk factors for trauma among those with prior disruptive behavior disorders. Those in rural settings with prior disorders had double the risk of those in urban settings with no prior disorders (odds ratio  =  2.35, 95% confidence interval 1.83–3.01). Similarly, those treated for five or more years had higher risks compared to those with no prior disorders (odds ratio  =  1.43, 95% confidence interval 1.26–1.63). Age at first diagnosis, number of specialist visits, and other neuropsychiatric comorbidities were not particularly ominous or reassuring (Table S1). The increased risk was apparent multiple years before the crash as measured either by time from birth or time before crash (Figure 2).

We found no evidence that the severity of injury was different in patients with disruptive behavior disorders. A total of 1,904 of the 3,421 trauma patients underwent surgery, with a rate similar for those with disorders and those without disorders (54% versus 56%, *p = *0.415). A total of 879 trauma patients required critical care treatment, with a rate similar for those with disorders and those without disorders (24% versus 26%, *p = *0.132). A total of 716 trauma patients stayed in hospital more than a week, with a rate similar for those with disorders and those without disorders (20% versus 21%, *p = *0.510). A total of 70 trauma patients died, with a rate similar for those with disorders and those without disorders (2.1% versus 2.0%, *p = *0.930).

## Discussion

We studied teenage male youth admitted to hospital for road trauma. We found high rates of disruptive behavior disorders evident years before the crash. Overall, attention deficit hyperactivity disorder, conduct disorder, and oppositional defiant disorder were associated with about a one-third increase in the risk of serious road trauma, which is similar in magnitude to the relative risk documented for individuals treated for epilepsy [67],[68]. Collectively, the attributable risk associated with these disorders explained about 1-in-20 crashes observed in this study. These findings were prevalent throughout the years, accentuated in rural settings, evident in the most severe cases, and difficult to attribute to chance.

One limitation in our study relates to the retrospective design. Teenagers and their families are aware of diagnoses, may self-restrict driving, and thereby attenuate all observed risks [69]. In addition, childhood psychiatric diagnoses can be mistaken and the misdiagnoses also bias our relative risk estimates toward the null [55],[70]. For example, if diagnostic sensitivity and specificity were each 95%, the true odds ratio would be about 2.0 rather than 1.3. Our controls, furthermore, were not immune to psychiatric illness, so that the attributable risk estimates are also conservative. If our study presumed a disease prevalence of 10% from population surveys [71], for example, the estimated odds ratio would be about 2.5 and the attributable risk would account for about 1-in-9 observed crashes.

Another large limitation in our study is that all patients diagnosed with disruptive behavior disorders had access to care and received treatment. Because care is effective, the observed increase in risk is smaller than would occur in patients with missed diagnoses, no access to care, or poor adherence to treatment. The Canadian setting also had multimodal social services during this interval devoted to educating families of affected children, adapting school programs for special needs, curbing alcohol drinking in youth, and subsidizing medications for children in poverty [72],[73]. If 50% of eligible patients received treatment and 50% of treated patients responded with positive benefits, for example, the true unmeasured baseline risk would equal an odds ratio of about 1.5 rather than 1.3.

A third limitation that causes our study to underestimate the association of disruptive behavior disorders with road trauma is that the data excluded girls [74]. To address this issue we retrieved the original databases, replicated our methods in girls rather than boys, and conducted a post hoc analysis. As anticipated, the results yielded a smaller sample (*n = *4,156) and about the same estimated risk (odds ratio 1.31, 95% confidence interval 1.07–1.61, chi-square  =  6.8, *p = *0.010). Hence, the association of disruptive behavioral disorders with road trauma extended to both teenage boys and girls. Of course, many issues remain for future research including medication level at time of injury, amount of driving, extent of brain trauma, and sequelae among those not hospitalized [75],[76].

Universal health care databases have strengths because they are the antithesis of small surveys using self-report. The sample size is substantial and represents a 100% response rate—thereby avoiding referral bias, selective participation, range restrictions, and other threats to validity. Outcomes reflect serious crashes and are not extrapolations based on surrogate tests of driving risk. Ascertainment of prior diagnoses is conducted in an objective manner blind to outcomes, free of recall bias, and comprehensive over a decade. The downside, however, is a lack of data from prospective observation of lifestyle, alcohol, drugs, speeding, distractions, impulsivity, undiagnosed internalizing disorders, and other mechanisms or mediators in the causal pathway to driver error [77].

The increased risk of road trauma associated with disruptive behavior disorders in male youth does not by itself justify withholding a driver's license. Many disorders can be treated effectively, so that well-managed patients could have outcomes similar to the population average [78],[79]. This study, as well, has no at-fault data so an alternative interpretation might be that such disorders merely impair a person's ability to avoid a mishap initiated by someone else. Our analysis could also be explained by a hidden third factor linked to both the disorder and crash risks; for example, undocumented head injuries. Most importantly, the observed increase in risk as pedestrians indicates that those who abstain from driving do not escape the danger of serious road trauma.

The strongest argument in favor of regulations is that bad driving imposes risks on other people and can destroy whole lives in a moment. The main rebuttal against regulations involves the reduced quality of life and increased workload for innocent individuals. Reporting by physicians of unfit drivers to vehicle licensing authorities is one policy option, particularly since the average patient in our study had multiple visits to a physician in the year before the trauma [80]. Regulations of psychiatric diagnoses, however, would be controversial given the unfair stigma and social discrimination that surrounds mental disorders [81]. A further caveat is that disruptive behavior disorders are sometimes overdiagnosed, open to debate, and could be abused by vehicle insurers [82],[83].

Roadway engineering is a different alternative for mitigating driver error by making the environment more forgiving. However, such well-intentioned policies sometimes lead to greater dangers for younger drivers [84]. A classic example involves designing the approach paths to road intersections with generous sight lines to accommodate drivers with slow reaction times (e.g., older drivers). This safety cushion, ironically, can tempt drivers who have quick reaction times to approach at faster speeds (e.g., younger drivers) [85]. As such, a policy of creating “forgiving roads” can ironically create “permissive roads” for those who are impulsive and have imperfect rule adherence. The underlying error is that drivers sometimes overestimate their skills and underestimate their risks [86].

The findings call attention to a widespread, preventable, and costly cause of death and disability. Specifically, disruptive behavior disorders could be considered as contributors to road trauma—analogous to seizure disorders, refraction errors, and some other medical diseases [17]. Greater attention by primary care physicians, psychiatrists, and community health workers might be helpful since interventions can perhaps reduce the risk including medical treatments (e.g., methylphenidate), avoidance of distractions (e.g., cell phone calls while driving), and basic practicalities (e.g., abstaining from alcohol). Most people know that teenage males are prone to traffic injuries, but the current data show that prevailing adjustments are not sufficient.

## Supporting Information

Table S1 **Technical appendix - additional analyses.**
(0.04 MB DOC)
