# Supplementary material for: Road Trauma in Teenage Male Youth with Childhood Disruptive Behavior Disorders: A Population Based Analysis
Source: PLoS Med. 2010 Nov 16;7(11):e1000369. doi: 10.1371/journal.pmed.1000369 (PMC2981585; doi:10.1371/journal.pmed.1000369)
Supplement: Table S1 — Technical appendix - additional analyses. (0.04 MB DOC) [file pmed.1000369.s001.doc]

| Table S1 Additional Specific Psychiatric Predictors of Trauma (n = 1431) † | | | | |  |
| --- | --- | --- | --- | --- | --- |
|  |  |  |  |  |  |
|  |  |  |  | Lower 95% | Upper 95% |
|  |  |  |  | Confidence | Confidence |
|  |  | Odds Ratio * |  | Interval | Interval |
|  |  |  |  |  |  |
| Age (per year increase) |  | 1.08 |  | 0.98 | 1.19 |
| Socio Economic Status (per quintile increase) | | 0.99 |  | 0.92 | 1.07 |
| Rural Home Location |  | 1.53 |  | 1.17 | 2.00 |
|  |  |  |  |  |  |
| Attention Deficit Hyperactivity Disorder (code 314) | | 1.02 |  | 0.83 | 1.26 |
| Other Disruptive Behavior Disorder (code 312, 313) | | 1.10 |  | 0.85 | 1.43 |
| Both Disorders |  | 1.10 |  | 0.88 | 1.37 |
|  |  |  |  |  |  |
| Age at First Psychiatric Viist |  | 0.99 |  | 0.95 | 1.03 |
| Total Years from First to Last Psychiatric Visit | | 1.03 |  | 1.00 | 1.07 |
| Age at Latest Psychiatric Visit |  | 1.03 |  | 0.99 | 1.07 |
| Total Number of Psychiatric Visits |  | 1.00 |  | 0.99 | 1.01 |
| Total Number of Specialist Psychiatrist Visits | | 1.00 |  | 0.99 | 1.01 |
| Years Since Last Psychiatric Visit to Admission | | 0.99 |  | 0.95 | 1.02 |
|  |  |  |  |  |  |
| Substance Abuse |  | 1.38 |  | 0.95 | 2.00 |
| Learning Disorder |  | 1.01 |  | 0.74 | 1.39 |
| Depression |  | 1.05 |  | 0.76 | 1.44 |
| Personality Disorder |  | 1.05 |  | 0.68 | 1.62 |
| Epilepsy |  | 1.50 |  | 0.77 | 2.92 |
| Movement Disorder |  | 1.00 |  | 0.76 | 1.32 |
| Mental Developmental Retardation |  | 0.86 |  | 0.42 | 1.78 |
|  |  |  |  |  |  |
|  |  |  |  |  |  |
|  |  |  |  |  |  |
|  |  |  |  |  |  |
|  |  |  |  |  |  |
|  |  |  |  |  |  |
|  |  |  |  |  |  |
|  |  |  |  |  |  |
|  |  |  |  |  |  |
|  |  |  |  |  |  |
| Footnote |  |  |  |  |  |
| * from univariate analysis |  |  |  |  |  |
| † analyses excludes those with no pyschiatric condition | | |  |  |  |
